# Analysis of bearing mechanism of large-diameter under-reamed piles based on model tests

**DOI:** 10.1371/journal.pone.0338899

**Published:** 2025-12-31

**Authors:** Jianhua Zhou, Yang Song, Bo Gong, Shuhai Liang, Heping Wang

**Affiliations:** 1 Civil Engineering College, Liaoning Technical University, Fuxin, China; 2 Railway Engineering College, Liaoning Railway Vocational and Technical College, Jinzhou, China; 3 Shen Kan Engineering & Technology Corporation, MCC, Shenyang, China; 4 College of Natural Resources Science and Technology, Xinjiang University of Technology, Hotan, China; Shuguang Hospital Affiliated to Shanghai University of Traditional Chinese Medicine, CHINA

## Abstract

Given the insufficient understanding of the bearing mechanism and failure modes of large-diameter under-reamed piles in complex strata, this study conducted scaled laboratory model tests based on similarity theory. A visualized “semi-model pile static loading-reaction frame” system was established to systematically investigate the influence of under-reaming angle (0° ~ 25°) and pile embedment depth (60 ~ 80 cm) on the bearing characteristics and failure mechanisms of the pile foundation. The results show that: 1) The under-reaming angle is the dominant factor controlling bearing performance. A reasonable increase in this angle can significantly enhance the ultimate bearing capacity, with a 204.99% improvement observed at 20° compared to the uniform-section pile. However, the enhancement effect weakens with increasing embedment depth. Comprehensive analysis suggests that 15° ~ 20° is the optimal under-reaming angle range; an excessive angle induces stress concentration at the “shoulder” of the pile, leading to interfacial detachment between pile and soil, thus limiting further improvement in bearing capacity. 2) The under-reamed structure effectively optimizes the load transfer path along the pile shaft. The end bearing resistance ratio increases first and then decreases with the under-reaming angle, reaching a maximum of 65.09% at 20°, indicating a transition of the load transfer mechanism from shaft-resistance-dominated to end-resistance-dominated behavior. 3) The failure morphology of the pile toe rock evolves from “penetrative shear failure” in the uniform pile (failure zone ≈ 1.25D) to “fan-shaped compaction” at the optimal under-reaming angle (≈ 4D), and further enlarging the angle results in unstable “bulging and collapse” failure. This study systematically reveals the full-process mechanism from load bearing to failure in large-diameter under-reamed piles, providing a theoretical basis for optimizing design parameters and predicting failure behavior. The findings offer valuable references for engineering design and improvement of design codes.

## 1 Introduction

As one of the most widely used deep foundation forms in geotechnical engineering, the vertical bearing performance of pile foundations plays a decisive role in the overall safety and stability of buildings and infrastructure. Under complex geological conditions, conventional uniform-section piles often suffer from insufficient end bearing resistance and limited deformation control capacity, making them difficult to meet the engineering requirements of high bearing capacity and low settlement. In contrast, under-reamed piles can significantly enhance vertical bearing capacity and overall stability by enlarging the pile toe bearing area and strengthening the pile-soil interlocking effect. Therefore, they have been widely applied in high-rise buildings, bridges, and soft soil regions [1]. However, the load transfer mechanism and failure modes of under-reamed piles with different under-reaming angles, embedment depths, and pile diameters have not yet been fully understood.

In terms of bearing mechanisms, Poulos and Randolph [2] theoretically analyzed the distribution characteristics of shaft resistance and end bearing resistance. He and Li et al. [3,4] employed numerical simulations combined with centrifuge model tests to investigate the influence of construction disturbance on the surrounding soil. Feng Guohui et al. [5] studied the effects of pile diameter and position on deformation patterns by considering pile-soil interaction. Nasr et al. [6] conducted a series of systematic model tests to investigate the mechanical response of single-finned piles in sand under vertical (V), torsional (T), and combined (T → V) loading conditions. By varying the fin length, width, shape, and number, they analyzed how these geometric parameters influence the maximum torsional bearing capacity of the piles. Zhang Lei et al. [7] and Zhao Chunfeng et al. [8] found through experiments that large-diameter under-reamed piles exhibit stronger deformation coordination, and their actual bearing capacity is often much higher than the traditional theoretical predictions. Xu Wei et al. [9] reported that rock-socketed under-reamed piles can fully mobilize end bearing resistance, thereby improving the overall stability of pile foundations.

Regarding settlement and deformation, Zhang Qianqing et al. [10] established a nonlinear settlement calculation method that accounts for soil softening along the pile shaft and hardening at the pile toe. Zhao Minghua et al. [11] modified the load transfer function to improve prediction accuracy. Vali Ramin [12] and Hossein [13] showed that variations in the embedment depth and enlargement ratio of the under-reamed section significantly influence settlement control and bearing performance. Hamderi [14] proposed an improved settlement estimation approach based on large-scale finite element models. Gao Denghui et al. [15] developed a constitutive model for pile-soil load transfer by considering the nonlinear stress-strain relationship and ultimate shear strength of the soil, and proposed a method for estimating settlement at arbitrary depths based on the total self-weight collapse value. Lin Chunjin et al. [16] verified the load transfer behavior between the pile toe and shaft soil through field experiments. Chen Li et al. [17] derived a practical single-pile settlement formula using the stress-area method. Gowthaman et al. [18] and Yang et al. [19] emphasized that combining three-dimensional numerical models with field monitoring can more accurately characterize group pile settlement behavior, providing a reliable approach for complex conditions. In another study, Nasr et al. [20] employed the PLAXIS 3D numerical analysis platform to examine the compressive behavior of under-reamed piles in stiff clay, focusing on the effects of enlargement diameter, spacing, and the number of enlarged bases. Their results indicated that these geometric factors have significant impacts on both the bearing capacity and failure patterns of the piles, with a triple-enlarged configuration increasing the bearing capacity by approximately 90%.

In terms of load transfer and failure modes, Dong Jinrong et al. [21] and Xu Xiankun et al. [22] revealed through field tests that the under-reamed section significantly enhances the contribution of end bearing resistance. Hu Qinghong et al. [23] found through combined experimental and numerical analyses that the failure of soil at the pile toe is the key factor determining the ultimate bearing capacity. Gao Guangyun et al. [24] demonstrated that removing sediment from the pile base can increase end bearing resistance, and that there exists an optimal matching range between pile length and enlargement ratio. Zhang Qi et al. [25] established a pile-soil interaction model and found that when D > 2d and the pile length increases beyond a threshold, the bearing capacity tends to level off. Harvey et al. [26], based on the “PISA” project, proposed a multi-spring analytical model to refine the pile-soil interaction at different depths, providing new theoretical insights for studying load transfer mechanisms. Wang Yifei et al. [27] further investigated the pile-soil interaction under seismic loading using MIDAS GTS NX finite element software, revealing the influence of dynamic loading on vertical load transfer behavior.

Overall, previous studies reveal that the bearing characteristics of under-reamed piles are jointly governed by the geometry of the pile toe, soil stratigraphy, and embedment conditions. Existing research has predominantly focused on predicting the static ultimate bearing capacity, modifying load-transfer functions, and validating numerical models. However, systematic model testing that captures the full-process evolution of load-bearing behavior in large-diameter under-reamed piles—particularly under different combinations of under-reaming angle and embedment depth—remains limited. In particular, the failure mechanisms of the soil at the pile toe, load-transfer pathways, and the coupled influence between under-reaming angle and embedment depth have not yet reached a unified understanding. Furthermore, most existing studies rely on numerical simulations or non-visual model tests, making it difficult to intuitively capture the deformation of the under-reamed section and the pile–soil interaction process.

To address these gaps, this study utilizes a visualized semi-model static loading system for under-reamed piles [28]. By systematically controlling two key parameters—the under-reaming angle and embedment depth—comparative model tests were performed to reveal the load-transfer mechanism of the pile–soil system, the evolution of end resistance, and the transition of failure modes. The objective is to fill the experimental research gap concerning the bearing mechanism and parameter-coupling effects of large-diameter under-reamed piles, thereby providing a scientific basis for design optimization and engineering applications.

## 2 Model test overview

### 2.1 Model test apparatus and semi-model principle

This study adopted a self-designed “semi-model pile static loading-reaction frame system,” which was constructed based on the mechanical symmetry principle. The geometric configuration, loading, and boundary constraints of the pile-soil system are symmetric along the pile axis, resulting in a symmetrical distribution of internal stress and strain fields. According to this principle, the complete three-dimensional pile-soil system is cut along the symmetric plane, and only one half is retained for experimental observation. This semi-model testing approach effectively reduces the model size while maintaining mechanical equivalence, and the use of a glass plate enables visualization of the pile-soil interaction process.

The test system mainly consists of four parts: a loading system, a reaction device, a monitoring system, and a data acquisition system. The loading system applies a vertical load using a hydraulic jack with a maximum capacity of 10 t, and a high-precision pressure sensor is used to monitor the load variation in real time. The settlement at the pile top is measured by a high-precision displacement meter, while the internal force distribution along the pile is obtained from resistance strain gauges arranged along the inner wall of the pile, enabling real-time monitoring throughout the test process.

The model box adopts a self-designed semi-cylindrical structure with internal dimensions of 100 cm in length (along the symmetric plane), 60 cm in radius, and 140 cm in height. To ensure ideal symmetric boundary conditions and minimize boundary effects, several key design measures were implemented:

(1) Friction reduction and visualization at the symmetric plane: A high-strength tempered glass panel was installed along the cut plane to serve as an observation window. A thin layer of silicone grease was applied to the inner surface of the glass and then covered with a plastic film to create a low-friction lubricated interface. This study adopted this well-established “silicone-grease + plastic-film” dual-layer friction-reduction treatment in physical model tests, aiming to markedly reduce the interface shear stress to a level far below the inherent strength of the soil so that it does not interfere with the main strain zone and failure mechanism around the pile. This treatment effectively approximates an ideal smooth symmetric boundary while simultaneously enabling direct visual observation of the pile–soil deformation and crack propagation processes [29,30].
(2) Model box size and boundary effect control: Previous studies have shown that when the box diameter is 2–3 times larger than the pile diameter, the test results are minimally affected by boundary effects [31]. Wang Xiaolong et al. [32] conducted semi-model tests on steel pipe piles under horizontal loading and found that the lateral soil displacement was mainly concentrated within a range of five pile diameters. To ensure that boundary effects can be neglected, the radial size of the model box (120 cm in diameter) was designed such that its ratio to the pile diameter (4 cm) reaches 30, and even relative to the maximum under-reamed diameter, the ratio exceeds 12—both far greater than the critical proportion reported in the literature. The box height (140 cm) is 1.75 times the pile length (80 cm), providing sufficient soil thickness beneath the pile toe to effectively simulate a semi-infinite medium, allowing the stress field and plastic zone to fully develop without constraint.

**Fig 1:**
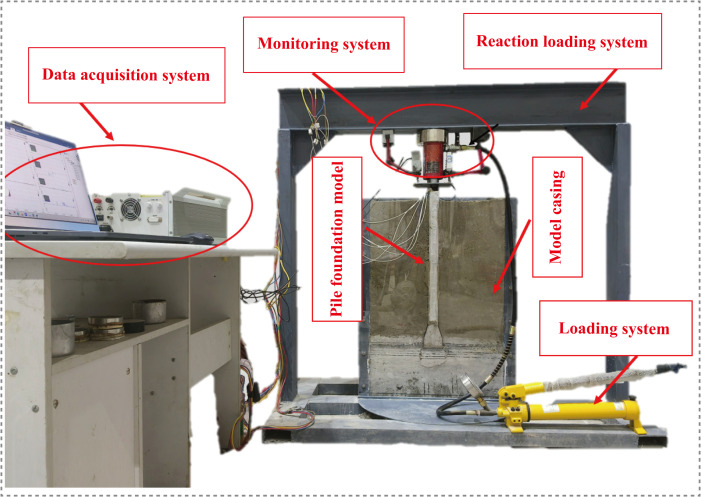
Experimental apparatus. (1) On-site sampling using drilling equipment. (2) Collected soil samples from the site.

The reaction frame adopts a portal steel structure, with both ends anchored to the laboratory concrete floor using high-strength bolts, providing a stable reaction foundation during loading. All sensor signals are connected to a static data acquisition system for automatic and synchronous measurement and storage, ensuring data accuracy and consistency. Liang Fayun and Yao Guosheng et al. [33,34] conducted semi-model pile tests to analyze pile behavior under lateral displacement and axial loading, confirming the reliability of the semi-model approach in studying pile mechanical responses. Therefore, the semi-model testing system adopted in this study not only enables visualization of the pile-soil interaction process but also ensures the accuracy of experimental data and the engineering relevance of the research findings through well-designed boundary conditions. Details are shown in Fig 1.

Compared with traditional full-model tests, the semi-model visualization loading system proposed in this study is established based on the mechanical symmetry principle of the pile-soil system. Using a symmetric-plane reaction device, the system reconstructs the full stress field equivalently while significantly reducing model size and minimizing boundary constraints. Meanwhile, transparent sidewalls enable real-time observation of soil deformation around the under-reamed section. This method maintains stress-field consistency while offering advantages such as reduced structural complexity, lower cost, and richer information acquisition. It thus provides an innovative experimental approach for investigating the bearing behavior and failure mechanisms of under-reamed piles.

### 2.2 Model similitude parameters

The validity of the pile foundation physical model test depends on strict similarity between the model and the prototype. In this study, based on similitude theory, a complete set of similitude ratios was established through dimensional analysis and derivation of the governing equations.

Considering the pile-rock interaction mechanism, twelve key physical quantities were selected. length *l*, density *ρ*, and stress *σ* were chosen as the basic physical quantities because their dimensions are independent and can be used to construct dimensionless functions *π* of all other physical variables. According to the second law of similitude, once the similitude ratios of these three fundamental quantities are determined, the similitude ratios of all other physical quantities can be uniquely defined.

According to the Buckingham *π* theorem, the dimensionless relationships among these variables can be expressed as:


f(l,u,A,ρ,σ,ε,E,ν,φ,C,F,P)=0 (1)

Therefore, once the similitude ratios of length *l*, density *ρ*, and stress *σ*, denoted as $C_{l}$、 $C_{\rho }$ and $C_{\sigma }$, are determined, the similitude ratios of all other quantities can be uniquely derived.

Both the model and the prototype must satisfy the differential equations of equilibrium in elasticity:


$$\begin{array}{c} \frac{\partial {\left(\sigma_{x}\right)}_{p}}{\partial x_{p}}+\frac{\partial {\left(\tau_{yx}\right)}_{p}}{\partial y_{p}}+\frac{\partial {\left(\tau_{zx}\right)}_{p}}{\partial z_{p}}+(f_{x}{)}_{p}=0\\ \frac{\partial {\left({\tau_{x}}_{y}\right)}_{p}}{\partial x_{p}}+\frac{\partial {\left(\sigma_{y}\right)}_{p}}{\partial y_{p}}+\frac{\partial {\left(\tau_{zy}\right)}_{p}}{\partial z_{p}}+(f_{y}{)}_{p}=0\\ \frac{\partial {\left({\tau_{x}}_{z}\right)}_{p}}{\partial x_{p}}+\frac{\partial {\left(\tau_{yz}\right)}_{p}}{\partial y_{p}}+\frac{\partial {\left(\sigma_{z}\right)}_{p}}{\partial z_{p}}+(f_{z}{)}_{p}=0 \end{array}\}$$ (2)

$$\begin{array}{c} \frac{\partial {\left(\sigma_{x}\right)}_{m}}{\partial x_{m}}+\frac{\partial {\left(\tau_{yx}\right)}_{m}}{\partial y_{m}}+\frac{\partial {\left(\tau_{zx}\right)}_{m}}{\partial z_{m}}+(f_{x}{)}_{m}=0\\ \frac{\partial {\left({\tau_{x}}_{y}\right)}_{m}}{\partial x_{m}}+\frac{\partial {\left(\sigma_{y}\right)}_{m}}{\partial y_{m}}+\frac{\partial {\left(\tau_{zy}\right)}_{m}}{\partial z_{m}}+(f_{y}{)}_{m}=0\\ \frac{\partial {\left({\tau_{x}}_{z}\right)}_{m}}{\partial x_{m}}+\frac{\partial {\left(\tau_{yz}\right)}_{m}}{\partial y_{m}}+\frac{\partial {\left(\sigma_{z}\right)}_{m}}{\partial z_{m}}+(f_{z}{)}_{m}=0 \end{array}\}$$ (3)

According to similitude theory, the following similitude relationships must exist between them:


$$\begin{array}{c} C_{l}=\frac{l_{p}}{l_{m}}=\frac{x_{p}}{x_{m}}=\frac{y_{p}}{y_{m}}=\frac{z_{p}}{z_{m}}\\ \;C_{\rho }=\frac{f_{p}}{f_{m}}=\frac{{\left(f_{x}\right)}_{p}}{{\left(f_{x}\right)}_{m}}=\frac{{\left(f_{y}\right)}_{p}}{{\left(f_{y}\right)}_{m}}=\frac{{\left(f_{z}\right)}_{p}}{{\left(f_{z}\right)}_{m}}\;\\ C_{\sigma }=\frac{\sigma_{p}}{\sigma_{m}}=\frac{{\left(\sigma_{x}\right)}_{p}}{{\left(\sigma_{x}\right)}_{m}}=\frac{{\left(\sigma_{y}\right)}_{p}}{{\left(\sigma_{y}\right)}_{m}}=\frac{{\left(\sigma_{z}\right)}_{p}}{{\left(\sigma_{z}\right)}_{m}}=\frac{{\left(\tau_{xy}\right)}_{p}}{{\left(\tau_{xy}\right)}_{m}}=\frac{{\left(\tau_{xz}\right)}_{p}}{{\left(\tau_{xz}\right)}_{m}}=\frac{{\left({\tau_{y}}_{z}\right)}_{p}}{{\left({\tau_{y}}_{z}\right)}_{m}} \end{array}\}$$ (4)

Combining Equation (3) and (4), one obtains:


$$\begin{array}{c} \frac{C_{\sigma }}{C_{l}}\frac{\partial {\left(\sigma_{x}\right)}_{m}}{\partial x_{m}}+\frac{C_{\sigma }}{C_{l}}\frac{\partial {\left(\tau_{yx}\right)}_{m}}{\partial y_{m}}+\frac{C_{\sigma }}{C_{l}}\frac{\partial {\left(\tau_{zx}\right)}_{m}}{\partial z_{m}}+C_{\rho }(f_{x}{)}_{m}=0\\ \frac{C_{\sigma }}{C_{l}}\frac{\partial {\left({\tau_{x}}_{y}\right)}_{m}}{\partial x_{m}}+\frac{C_{\sigma }}{C_{l}}\frac{\partial {\left(\sigma_{y}\right)}_{m}}{\partial y_{m}}+\frac{C_{\sigma }}{C_{l}}\frac{\partial {\left(\tau_{zy}\right)}_{m}}{\partial z_{m}}+C_{\rho }(f_{y}{)}_{m}=0\\ \frac{C_{\sigma }}{C_{l}}\frac{\partial {\left({\tau_{x}}_{z}\right)}_{m}}{\partial x_{m}}+\frac{C_{\sigma }}{C_{l}}\frac{\partial {\left(\tau_{yz}\right)}_{m}}{\partial y_{m}}+\frac{C_{\sigma }}{C_{l}}\frac{\partial {\left(\sigma_{z}\right)}_{m}}{\partial z_{m}}+C_{\rho }(f_{z}{)}_{m}=0 \end{array}\}$$ (5)

By combining Equation (1) and (5), the following relationship is obtained:


$$\frac{C_{\sigma }}{C_{l}C_{\rho }}=1,\;C_{\sigma }=C_{l}C_{\rho }$$ (6)

Equation (6) serves as the core equation linking the three fundamental similitude ratios. In this test, the geometric similitude ratio was set as $C_{l}=1/50$. To accurately simulate the self-weight stress field, the same material as the prototype was used, i.e., $C_{\rho }=1$. Substituting these values into Equation (6), the stress similitude ratio $C_{\sigma }=1/50$ can be determined. Based on this, all similitude constants derived from dimensional analysis are summarized in Table 1.

**Table 1:** Similitude constants for model test.

Physical quantity	Parameter	Similitude relation	Similitude ratio
Length	*l*	*C*_*l*_ * = l*_*m*_ */l*_*p*_	1:50
Density	*ρ*	*C*_*ρ*_* = *1	1:1
Displacement	_*δ*_	*C*_*δ*_*/C*_*l*_* = *1	1:50
Stress	*σ*	*C*_*σ*_*/*(*C*_*ρ*_*C*_*l*_)*=*1	1:50
Strain	*ε*	*C*_*ε*_* = *1	1:1
Elastic modulus	*E*	*C*_*E*_*/*(*C*_*ρ*_*C*_*l*_)*=*1	1:50
Poisson’s ratio	*μ*	*C*_*μ*_* = *1	1:1
Internal friction angle	*φ*	*C*_*φ*_* = *1	1:1
Cohesion	*c*	*C*_*c*_*/*(*C*_*ρ*_*C*_*l*_)*=*1	1:50

### 2.3 Model test soil

**Fig 2:**
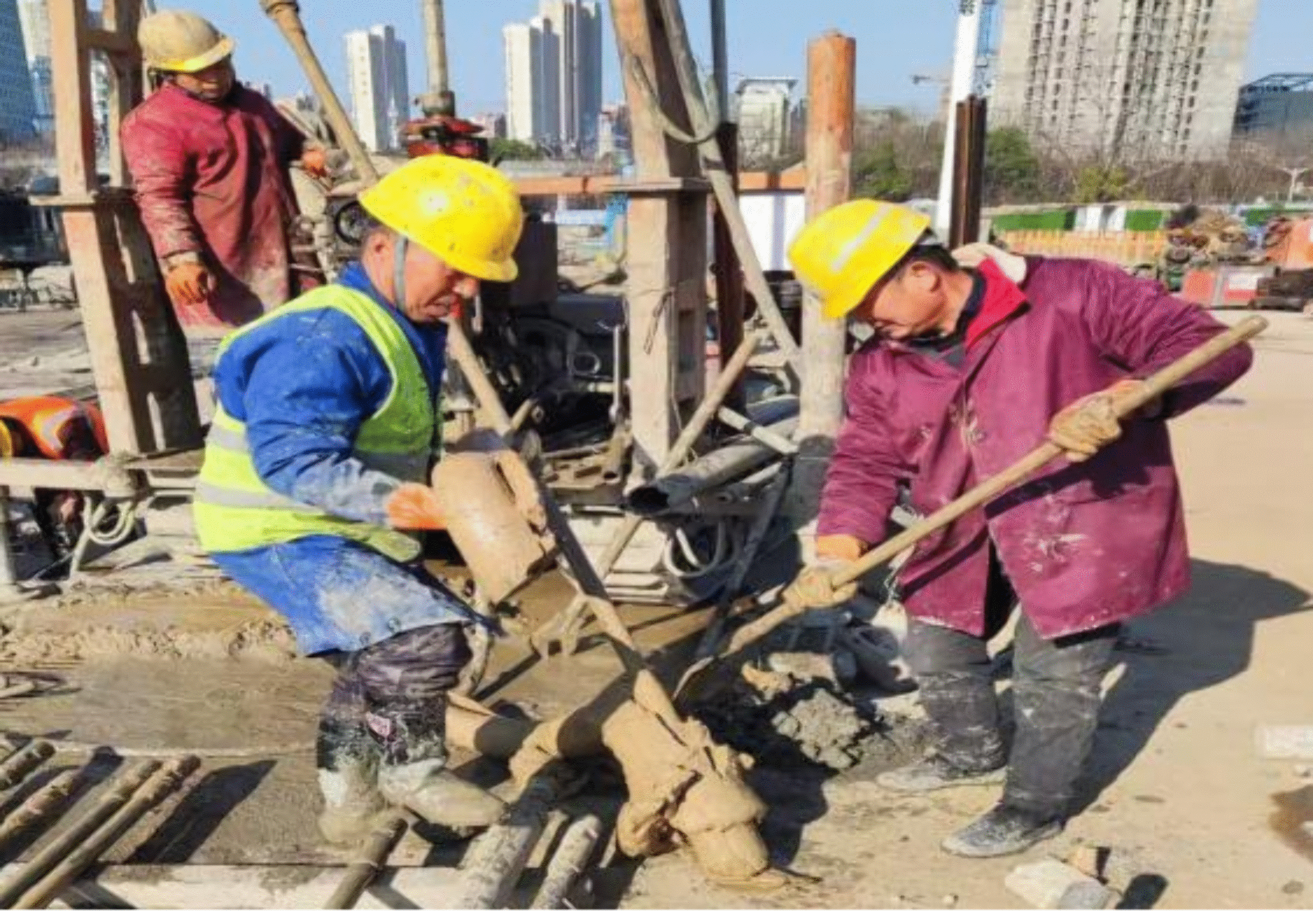
On-site soil sampling.

To ensure the accuracy of the experimental results, undisturbed soil sampling was first carried out at the construction site. (Fig 2) A drilling machine was used to collect samples from zones where the soil layers were relatively homogeneous and free of impurities. Site stratigraphic investigation revealed that the strata mainly consist of miscellaneous fill, silty clay, and slightly weathered bedrock. Considering both the stratigraphic characteristics and the feasibility and representativeness of the physical model tests, a reasonable simplification of the complex stratigraphic sequence was performed. Ultimately, the clay layer and the weathered rock layer were selected as representative strata for the experiment.

After sampling, protective measures were immediately applied to prevent exposure and maintain moisture, and the samples were promptly transported to the laboratory for physical and mechanical property testing. The tests determined the fundamental parameters—including density, moisture content, void ratio, and strength indices—which provided a reliable basis for the preparation of analogous materials used in subsequent model tests.(see Table 2 for details).

**Table 2:** Basic parameters of in-situ soil layers.

Soil layer	Unit weight(kN/m^3^)	Elastic modulus *E*(MPa)	Cohesion *C*(kPa)	Internal friction angle φ(°)
Silty clay layer	19.8	21.5	24.2	18.8
Slightly weathered rock	25.4	48.8	95.6	31.6

Based on the physical parameters obtained from the undisturbed soil samples, analogous materials were prepared and verified according to similarity theory. In this study, the geometric similarity ratio of the model was set to 1:50, and the corresponding mechanical parameter scaling relationships were determined according to the similarity theorem. The similarity ratios for stress, displacement, elastic modulus, and cohesion were all 1:50 [35].

Considering the actual geological conditions, quartz sand was selected as the aggregate, gypsum and cement were used as cementing agents, and barite powder was added as a weighting material to adjust the unit weight. A small amount of glycerol was mixed into the water to improve the workability and wetting properties of the mixture [36,37].

Based on this material system, multiple mix proportion tests were conducted. The density, elastic modulus, cohesion, and internal friction angle of each sample group were determined through direct shear and uniaxial compression tests. The optimal mix proportions were then selected to ensure that the mechanical parameters of the model materials closely matched the scaled target values [38,39].

The final analogous soil properties were found to be highly consistent with the target parameters, indicating that the selected proportions satisfied the similarity requirements. All mix proportions were expressed by mass ratio (see Table 3 for details).

**Table 3:** Basic parameters of analogous soil layer proportions.

Soil layer	Sand-binder ratio	Gypsum-cement ratio	Unit weight(kN/m^3^)	Elastic modulus *E*(MPa)	Cohesion *C*(kPa)	Internal friction angle φ(°)
Model clay	11:1	1.2:1	18.5	0.42	0.5	18.4
Model weathered rock	3:1	1:4	23.5	1.03	2.0	30.8

### 2.4 Model test scheme

#### 2.4.1 Model pile design.

**Fig 3:**
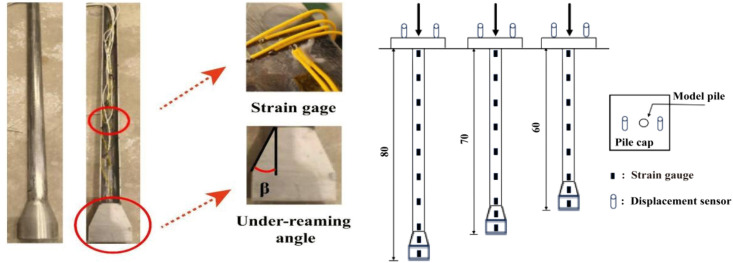
Layout of strain measuring points on the model pile. (1) *β* = 0° (2) *β* = 5°. (3) *β* = 10°. (4) *β* = 15.° (5) *β* = 20°. (6) *β* = 25°.

According to the strength similarity criterion and a geometric scale ratio of 1:50, aluminum tubes were selected as the material for the model piles. The mechanical properties of the aluminum tube exhibit good consistency with those of actual concrete piles [40]. To accurately measure the internal force distribution along the pile shaft, strain gauges were installed inside the pile wall at intervals of 10 cm from the pile top downward, with an additional measurement point placed 5 cm above the pile toe. (see Fig 3 for details).

Before attaching the strain gauges, the surface was polished and cleaned to remove dust. The gauges were initially fixed with 502 adhesive, then covered with a layer of epoxy resin for protection, and finally sealed with paraffin wax to improve insulation, moisture resistance, and long-term stability. All strain gauges, together with the data acquisition system, were calibrated under standard laboratory conditions (20 ± 2 °C) to ensure the accuracy of the gauge factors. To minimize the influence of temperature fluctuations on the test results, temperature self-compensated strain gauges were adopted, and all signal cables were securely bundled to suppress noise caused by wire movement. All wires were numbered for identification, assembled in sequence, and tested for electrical continuity to ensure reliable data acquisition.

The model soil was prepared based on the determined similarity ratio and mix proportion parameters. A layered filling technique was adopted, with each layer controlled to a thickness of approximately 5 cm and compacted to ensure uniform density. During installation, the model pile was kept vertical and accurately embedded in the pre-set position, ensuring that the pile axis coincided precisely with the centerline of the model box.

#### 2.4.2 Loading scheme.

To ensure scientific validity and comparability, the loading procedure followed the Code for Testing of Building Foundation and Pile Foundations (DBJ/T 15-60-2019), adopting a slow stepwise loading method for single-pile vertical compression tests. The estimated maximum load was divided into several equal increments, each of 1 kN. After each load step was applied, it was maintained for 10 ~ 15 minutes until the pile top displacement rate stabilized before proceeding to the next stage.

Upon completion of loading, unloading was carried out step by step in reverse order until the load was completely removed, and the residual settlement and rebound deformation were recorded.

In the scaled indoor model tests, the pile diameter was set to 4 cm, while pile lengths (Embedment depth *h*)were 60 cm, 70 cm, and 80 cm. The under-reaming angles (*β*) were designed as 0°, 5°, 10°, 15°, 20°, and 25°, covering six working conditions to systematically investigate the influence of under-reaming angle and pile length on bearing performance. The specific parameters are listed in Table 4.

**Table 4:** Model pile test scheme.

Pile No.	Pile diameter D(cm)	Under-reaming angle *β*(°)	Embedment depth *h*(cm)
1#	4	0	60
2#		5	60
3#		10	60
4#		15	60
5#		20	60
6#		25	60
7#		15	70
8#		15	80
9#		20	70
10#		20	80

## 3 Results and analysis

### 3.1 Failure mode analysis of model piles

**Fig 4:**
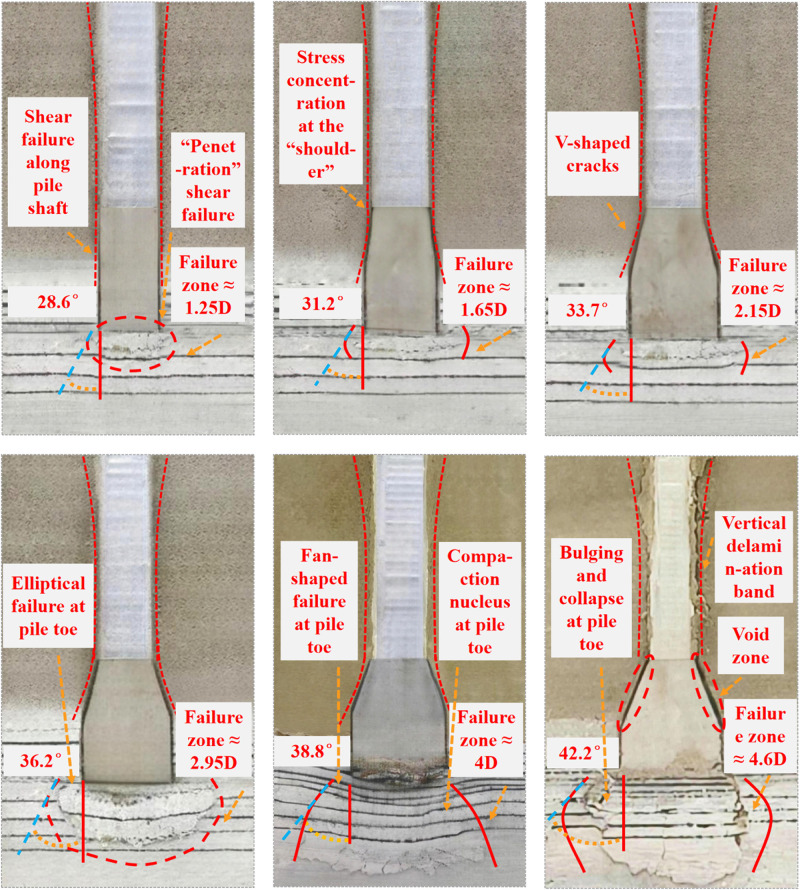
Failure patterns of model piles with different under-reaming angles.

To compare the ultimate failure mechanisms of model piles with different under-reaming angles, this section summarizes the crack distribution, rupture angle, and failure morphology for each test condition, and presents typical failure diagrams of model piles (see Fig 4).

A total of ten test conditions were designed in this study, among which six representative cases with under-reaming angles of 0°, 5°, 10°, 15°, 20°, and 25° were selected for detailed analysis. These cases cover the main failure modes from uniform-section piles to large under-reamed piles, providing a systematic understanding of how variations in the under-reaming angle influence the failure morphology at the pile toe and the evolution of the rupture angle.

Under the condition of *β* = 0°, the rock mass beneath the pile toe initially experienced an elastic compression stage during loading. When the applied load increased to approximately 3.08 kN, the stress at the pile toe exceeded the shear strength of the rock mass, leading to the initiation of microcracks in the high-stress region below the pile toe. These cracks gradually propagated upward and outward, eventually coalescing into a continuous shear rupture surface extending to the ground surface. The cracks exhibited large apertures and distinct residual openings, which were difficult to close after unloading. The failure zone beneath the pile toe was confined within about 1.25D (1.25 times the pile diameter), with a rupture angle of approximately 28.6°, representing a typical “penetration-type” shear failure (refer to the failure pattern in which the pile toe is directly pressed into the bedrock, and the failure zone is concentrated around the pile toe). This case exhibited the lowest bearing capacity among all test conditions.

When *β* increased to 5°, microcracks began to appear at a load of 4.09 kN. Due to the presence of the enlarged base, the load transfer path changed. Stress concentration occurred at the “shoulder” region — the junction between the under-reamed section and the uniform shaft. Cracks first initiated at this location and then propagated under the combined action of vertical shearing and horizontal compression. As the load continued to increase, the failure pattern evolved from the “localized” mode of the uniform-section pile to a more outwardly expanded pattern. The failure zone extended to about 1.65D, with a rupture angle of 31.2°, and the pile-tip resistance was higher than that of the uniform-section pile.

At *β* = 10°, noticeable crushing occurred at the “shoulder” when the load reached 6.02 kN. Cracks expanded across the entire under-reamed section and extended upward to the pile shaft. These cracks gradually interconnected, forming shear bands and producing characteristic “V-shaped” fissures in the under-reamed section (refers to the inverted-V-shaped tensile cracks developing downward on both sides of the under-reamed shoulder). The failure zone extended to approximately 2.15D, and the rupture angle increased to 33.7°. At this stage, a synergistic effect between tip resistance and shaft friction was established.

When *β* increased to 15°, microcracks in the rock mass beneath the pile toe appeared only after the load reached 9.04 kN. No obvious stress concentration occurred at the “shoulder.” During most of the loading process, the pile-soil system remained in a stable and coordinated compression state, and macroscopic cracks appeared only in the later loading stage. The failure zone extended to approximately 2.95D, with a rupture angle of 36.2°, showing a regular “elliptical” failure pattern. The contribution of tip resistance increased significantly, leading to a marked improvement in bearing capacity.

When *β* reached 20°, the failure mechanism was similar to that of the 15° case, characterized by progressive deep compaction failure. In the later loading stage, a dense “compression core” formed beneath the pile toe. Vertical cracks in the uniform shaft were only partially connected, most being short and moderately open with minimal residual deformation. “V-shaped” cracks appeared symmetrically on both sides of the under-reamed section. The failure zone extended to about 4D, and the rupture angle increased to 38.8°, forming a distinctive “fan-shaped” failure pattern (refers to the fan-shaped compacted settlement zone extending outward from the lower edge of the pile base). The coordination between tip resistance and shaft friction was optimal, and both bearing capacity and stability reached their best performance.

At *β = *25°, the failure mechanism changed significantly. The excessively large under-reaming angle intensified the stress concentration at the “shoulder,” causing local crushing and circumferential detachment cracks to appear during the early loading stage. This detachment led to a loss of contact between the pile and the surrounding soil, preventing the effective mobilization of shaft resistance. Subsequently, the cracks propagated upward, forming vertical separation zones extending to the pile top. The cracks exhibited wide openings and remained unclosed after unloading. Although the failure range expanded to 4.6D and the rupture angle increased to 42.2°, the failure mode transitioned from a regular “fan-shaped” pattern to an irregular “bulging-collapse” failure (refers to the process in which the local soil at the pile toe first bulges and then collapses, forming an arc-shaped failure band). The excessive under-reaming angle caused pile-soil separation, disrupted the cooperative mechanism between tip and side resistance, and ultimately reduced the overall bearing performance.

**Fig 5:**
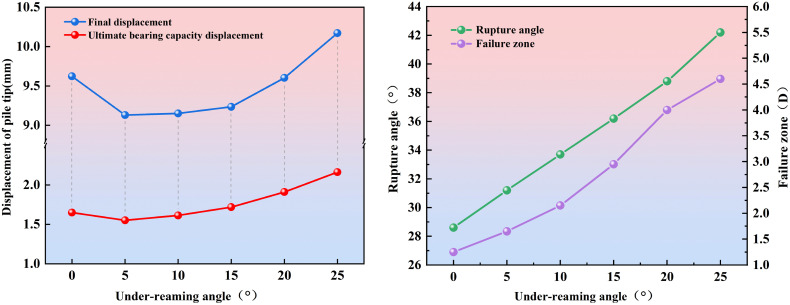
Evolution of failure degree of model piles with different under-reaming angles.

To quantitatively describe the failure characteristics under different under-reaming angles, the settlements corresponding to the ultimate bearing capacity and those at the end of failure were plotted, as shown in Fig 5. It can be observed that the settlement of the under-reamed pile decreases first and then increases with increasing under-reaming angle.

Compared with a straight-shaft pile, the inclusion of an enlarged base significantly reduces settlement; however, when the under-reaming angle increases from 10° to 20°, the settlement increment at failure increases by 0.92% and 3.98%, respectively. When the angle further increases from 20° to 25°, the increment rises to 5.91%. This pronounced increase steepens the settlement-angle curve, indicating that both the local failure strength and the extent of the failure zone at the pile toe become significantly greater.

From the perspective of rupture angle and failure extent, as the under-reaming angle increases from 0° to 25°, the increments in rupture angle are 9.09%, 8.01%, 7.42%, 7.18%, and 8.76%, respectively, showing a trend of “gradual decrease followed by renewed increase.” In particular, the increment rises again between 20° and 25°, suggesting more pronounced stress concentration at the pile toe within this interval. Meanwhile, as the under-reaming angle increases from 0° to 25°, the increments in the failure extent are 32%, 30.30%, 37.21%, 35.59%, and 15%, respectively. The increments generally remain around 30%, but when the angle further increases to 25°, they abruptly drop to 15%. This sudden decrease is mainly attributed to the transition of the failure mechanism at 25° from a “fan-shaped compaction mode” to a “bulging-collapse mode,” in which the soil within the failure zone exhibits outward bulging accompanied by partial collapse. As a result, the expansion of the failure extent becomes more constrained by the local yielding of the soil.

### 3.2 Settlement characteristics of under-reamed piles

**Fig 6:**
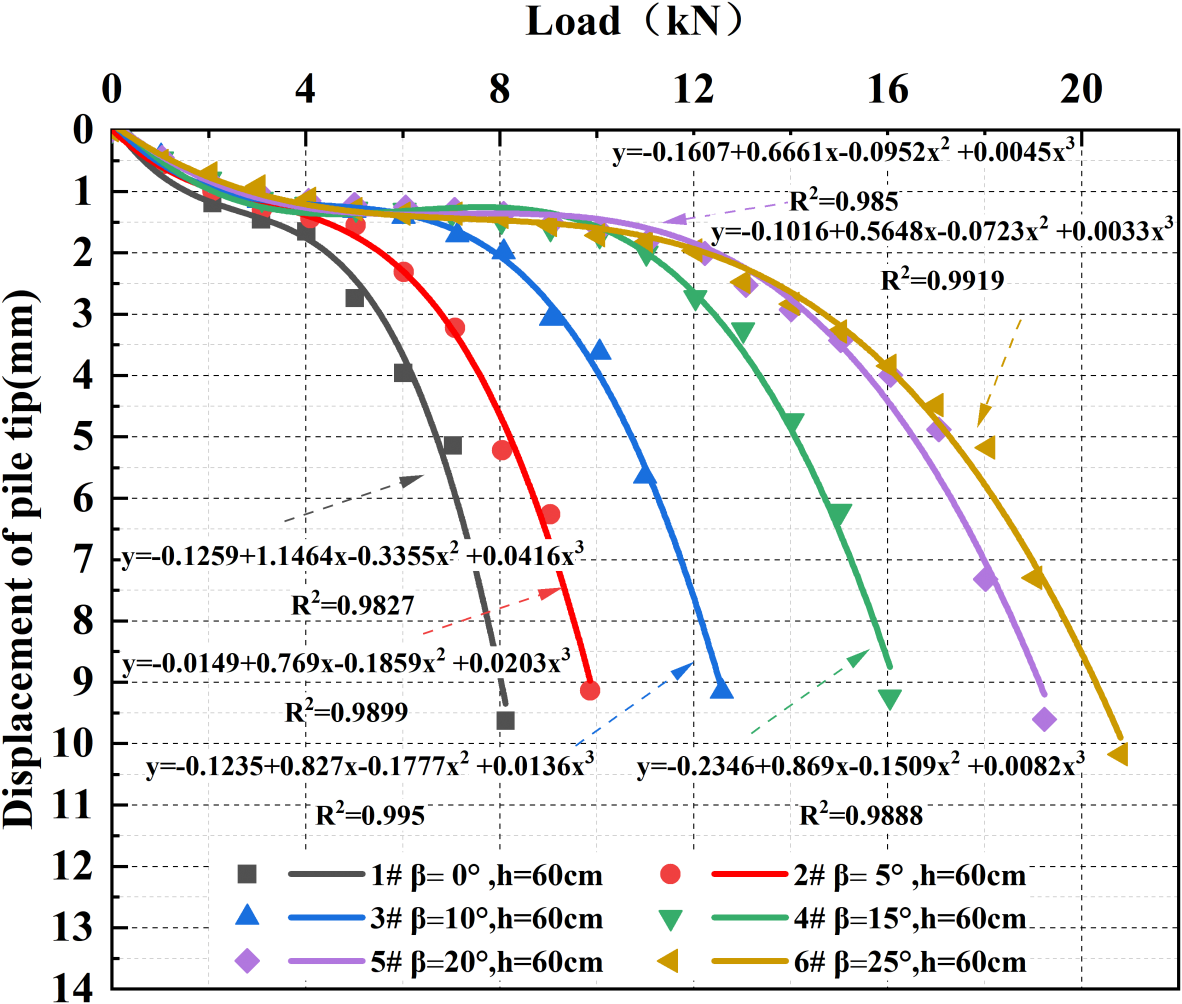
Load-displacement curves of piles under different under-reaming angles.

Based on the experimental results of piles with a total length of 60 cm under different under-reaming angles, the load-displacement curves for each working condition were organized and fitted, as shown in Fig 6. The coefficients of determination (R^2^) of the fitted curves ranged from 0.97 to 0.99, indicating high fitting accuracy.

As illustrated in Fig 6, under the same pile diameter and embedded depth, the load-displacement curves of the model piles can generally be divided into three characteristic stages: the initial compression stage, the steady load transfer stage, and the ultimate failure stage.

In the initial compression stage, the applied load is relatively small, and the bearing stratum beneath the pile toe is mainly in an elastic deformation state. The rock mass at the pile base is gradually compacted, leading to a slow increase in settlement, and the bearing capacity has not yet been fully mobilized.

During the steady load transfer stage, the shaft resistance of the pile begins to develop gradually, and a new mechanical equilibrium is established within the pile-soil system. The load increment is mainly carried by the shaft resistance, and the settlement curve tends to become gentler.

As the load continues to increase, once the load transmitted to the pile toe exceeds the shear strength of the bearing layer, the rock mass structure at the pile base fails and shear slip occurs, marking the onset of the ultimate failure stage. At this point, additional loading primarily induces plastic deformation and structural failure in the pile base, the settlement rate rises sharply, and a distinct steep segment appears on the curve, indicating that the pile has reached its ultimate bearing capacity.

The results indicate that the under-reaming angle has a significant influence on the evolution of the load-displacement curves. As the under-reaming angle increases from 0° to 25°, the duration of the steady load transfer stage progressively extends, and the settlement rate at the pile top decreases markedly, implying that a larger proportion of the vertical load is carried by the pile base. In other words, a longer steady stage corresponds to a higher ultimate bearing capacity.

Taking a settlement of 1.5 mm as a reference point, the bearing capacities of piles with under-reaming angles of 0°, 5°, 10°, 15°, 20°, and 25° were 4.86 kN, 6.19 kN, 7.63 kN, 10.07 kN, 10.56 kN, and 9.51 kN, respectively—representing increases of 27.37%, 57.00%, 107.20%, 117.28%, and 95.68% compared with the straight pile (*β = *0°).

It is evident that increasing the under-reaming angle enhances the vertical bearing capacity of the pile. However, when *β* exceeds 25°, the rate of improvement decreases. This observation is consistent with the failure mode analysis in Section 3.1, where an excessively large under-reaming angle was found to induce severe stress concentration in the “shoulder” region, forming localized “void zones” that weaken the cooperative deformation of the pile-soil interface and limit the mobilization of end bearing resistance. Therefore, an under-reaming angle of 20° is considered the most favorable for coordinated load transfer and deformation of the pile-soil system.

### 3.3 Analysis of ultimate bearing capacity of under-reamed piles

For the working condition with *β *= 0°, i.e., the straight pile, the loading test serves as the reference group for comparison with under-reamed piles. At the initial stage of loading, the load-displacement curve exhibits a steep descending trend. As the vertical load increases, the end bearing resistance of the pile gradually develops. When the load reaches 4.01 kN, a distinct inflection point appears, corresponding to a pile-soil relative displacement of 1.65 mm, which indicates that the pile has reached its ultimate bearing capacity. With further loading, the curve shows a pronounced descending segment, signifying that shear failure occurs in the soil at the pile toe.

**Fig 7:**
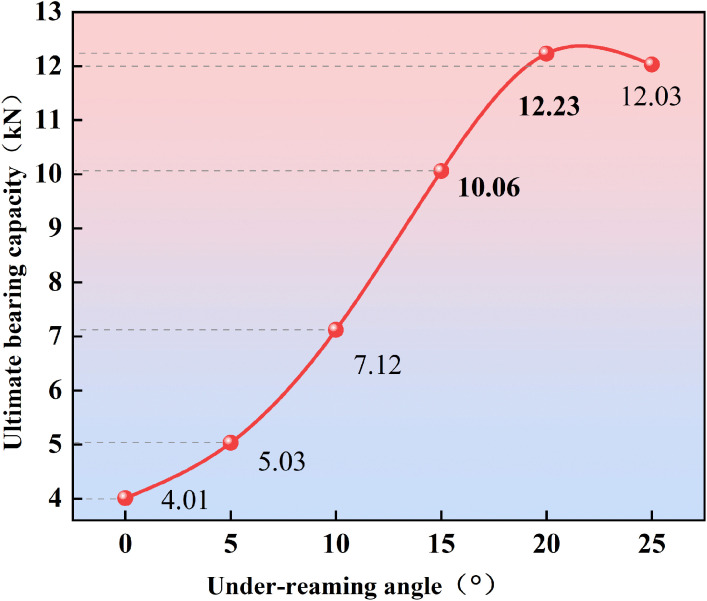
Relationship between ultimate bearing capacity and under-reaming angle.

The relationship between ultimate bearing capacity and under-reaming angle is plotted in Fig 7. Compared with the straight pile, piles with under-reaming angles of 5° and 10° exhibit ultimate bearing capacities of 5.03 kN and 7.12 kN, respectively—representing increases of 25.44% and 77.56%. However, the enhancement remains relatively modest at these smaller angles. When the under-reaming angle increases to 15°, 20°, and 25°, the ultimate bearing capacity rises significantly to 10.06 kN, 12.23 kN, and 12.03 kN, corresponding to increases of 150.87%, 204.99%, and 200.00%, respectively.

Among these, the under-reaming angle *β *= 20° achieves the maximum bearing capacity, indicating an optimal balance between load transfer and stress distribution. Although the 25° pile still shows higher bearing capacity than the straight pile, its improvement slightly declines, suggesting that excessive enlargement of the under-reamed base leads to stress concentration and localized detachment near the shoulder region, reducing the efficiency of end bearing resistance mobilization.

### 3.4 Analysis of embedment depth of under-reamed piles

**Fig 8:**
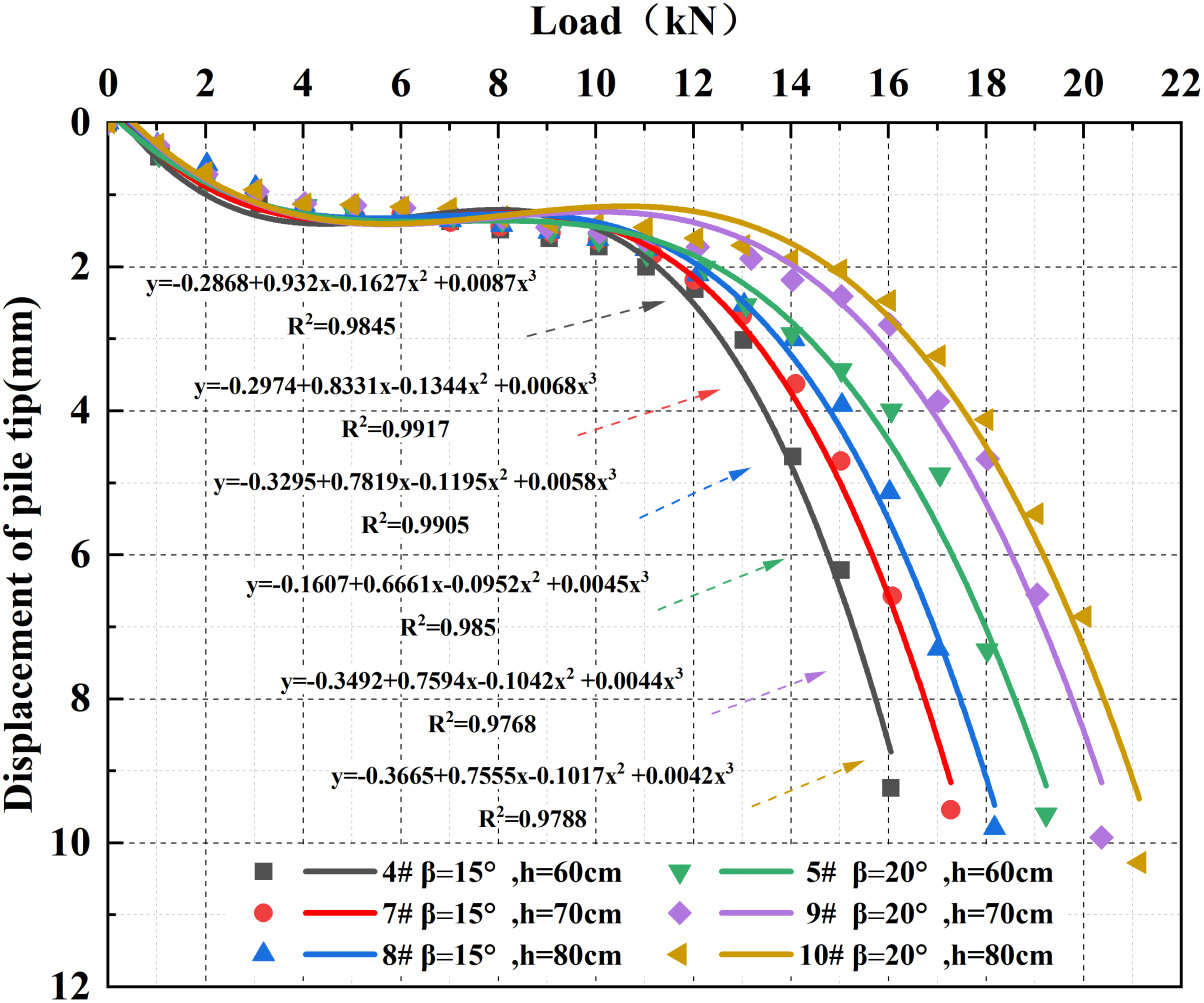
Load-displacement curves under different under-reaming angle and embedment depth conditions.

Based on the test results of under-reamed piles with 15° and 20° base angles under different embedment depths, the corresponding load-displacement curves were plotted, as shown in Fig 8. The results indicate that when the under-reaming angle remains constant, increasing the embedment depth can effectively improve the ultimate bearing capacity of the pile foundation.

For the 15° under-reamed pile, the ultimate bearing capacities at embedment depths of 60 cm, 70 cm, and 80 cm are 10.06 kN, 11.19 kN, and 12.09 kN, corresponding to increases of 11.23% and 8.04%, respectively. For the 20° under-reamed pile, the ultimate bearing capacities at the same depths are 12.23 kN, 13.19 kN, and 14.01 kN, with respective increases of 7.85% and 6.22%. In both cases, the rate of improvement in ultimate bearing capacity from 70 cm to 80 cm is smaller than that from 60 cm to 70 cm, by 3.19% for the 15° pile and 1.63% for the 20° pile. These results indicate that while increasing embedment depth effectively enhances bearing capacity, the marginal gains gradually decrease.

Further comparison shows that when the under-reaming angle increases from 15° to 20°, the ultimate bearing capacities at embedment depths of 60 cm, 70 cm, and 80 cm increase by 21.57%, 17.87%, and 13.70%, respectively. This demonstrates that under identical variations in under-reaming angle, the effect of increasing embedment depth on bearing capacity becomes less significant as depth increases. Therefore, in practical engineering design, the synergistic influence between under-reaming angle and embedment depth should be comprehensively considered to achieve an optimal balance between bearing capacity and constructability.

### 3.5 Verification and discussion of the ultimate bearing capacity determination method

**Fig 9:**
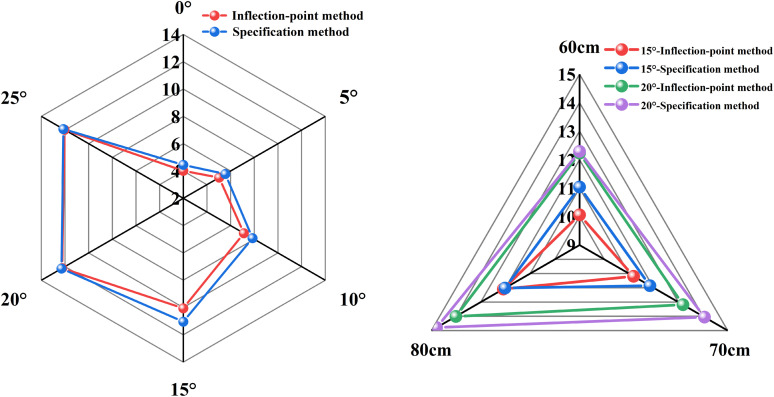
Comparison of ultimate bearing capacities obtained from the inflection-point method and the displacement-based code method under different under-reaming angle and embedment depth conditions.

To enhance the standardization and engineering comparability of the ultimate bearing capacity determination for under-reamed piles, this study adopts the displacement-based criterion recommended in the Technical Code for Testing of Building Foundation Piles (JGJ 106–2014), in addition to the “inflection-point method” used to identify the characteristic load on the load-settlement curve [41]. According to the code, for large-diameter piles with a base diameter D ≥ 800 mm, the ultimate bearing capacity is defined as the load corresponding to a settlement of *s* = 0.05D. Based on the geometric similitude ratio of the model test (1:50), the prototype diameter of the under-reamed pile used in this study satisfies this requirement, yielding a model-scale settlement criterion of *s* = 2 mm. Accordingly, the load corresponding to *s* = 2 mm on the model load-settlement curve was extracted as the standardized ultimate bearing capacity, and the results were compared with those obtained from the inflection-point method, as shown in Fig 9.

For under-reaming angles of 0°, 5°, 10°, 15°, 20°, and 25°, the displacement-based method yielded ultimate capacities of 4.45 kN, 5.55 kN, 7.85 kN, 11.04 kN, 12.29 kN, and 12.13 kN, respectively. In comparison, the relative error between the two methods ranged from a minimum of 0.49% to a maximum of 9.89%. For different embedment depths, the error varied between 0.58% and 6.19%. It is worth noting that the inflection-point method generally produced slightly lower values than the code method. This is mainly because a conservative criterion was adopted herein, whereby the load level immediately preceding the identified inflection point was taken as the ultimate capacity. Despite this conservatism, the two approaches show good overall agreement and consistently capture the significant transition in the load-carrying behavior. In summary, the inflection-point method effectively and reliably identifies the abrupt change in bearing capacity in the present tests, and its results are validated by the code-based method, demonstrating its suitability for analyzing the load-bearing characteristics of the model under-reamed piles.

### 3.6 Empirical model for predicting bearing capacity

Mechanically, the under-reaming angle (*β*) governs the effective load-bearing area and the stress-dispersion angle at the pile base, whereas the embedment depth (*ℎ*) controls the confinement and the stress-dispersion path within the surrounding soil. The two variables therefore exert a coupled influence on the ultimate bearing capacity, making it difficult to construct an explicit functional relationship based on a single variable. For this reason, *β* and *h* were selected as the core independent variables for developing the predictive model. Initial attempts using conventional nonlinear polynomial regression revealed that higher-order polynomials were unable to stably capture the strong coupling between *β* and *h*. These models exhibited high sensitivity and required numerous regression coefficients, which reduced interpretability.

To provide a more rational representation of the underlying mechanisms, an integrated treatment of *β* and *h* was adopted, whereby compound terms reflecting their coupling effect were introduced. Through multiple rounds of model testing, the term β/h was incorporated to characterize the relative variation between the under-reaming angle and the embedment depth, while the enhanced interaction term ${\left(\beta h\right)}^{n}$ was added to account for their synergistic amplification. This led to the development of the following two-parameter nonlinear regression model:


$$F=a_{0}+a_{1}\left(\frac{\beta }{h}\right)+a_{2}{\left(\beta h\right)}^{n}$$ (7)

where F is the ultimate bearing capacity (kN), and $a_{0}$、 $a_{1}$、 $a_{2}$ and n are regression coefficients. The fitted formula obtained via Origin regression analysis is as follows:


$$F=\text{3}\mathrm{.624}-4\mathrm{.787}\left(\frac{\beta }{h}\right)+\text{0}\mathrm{.014}{\left(\beta h\right)}^{0.915}$$ (8)

**Fig 10:**
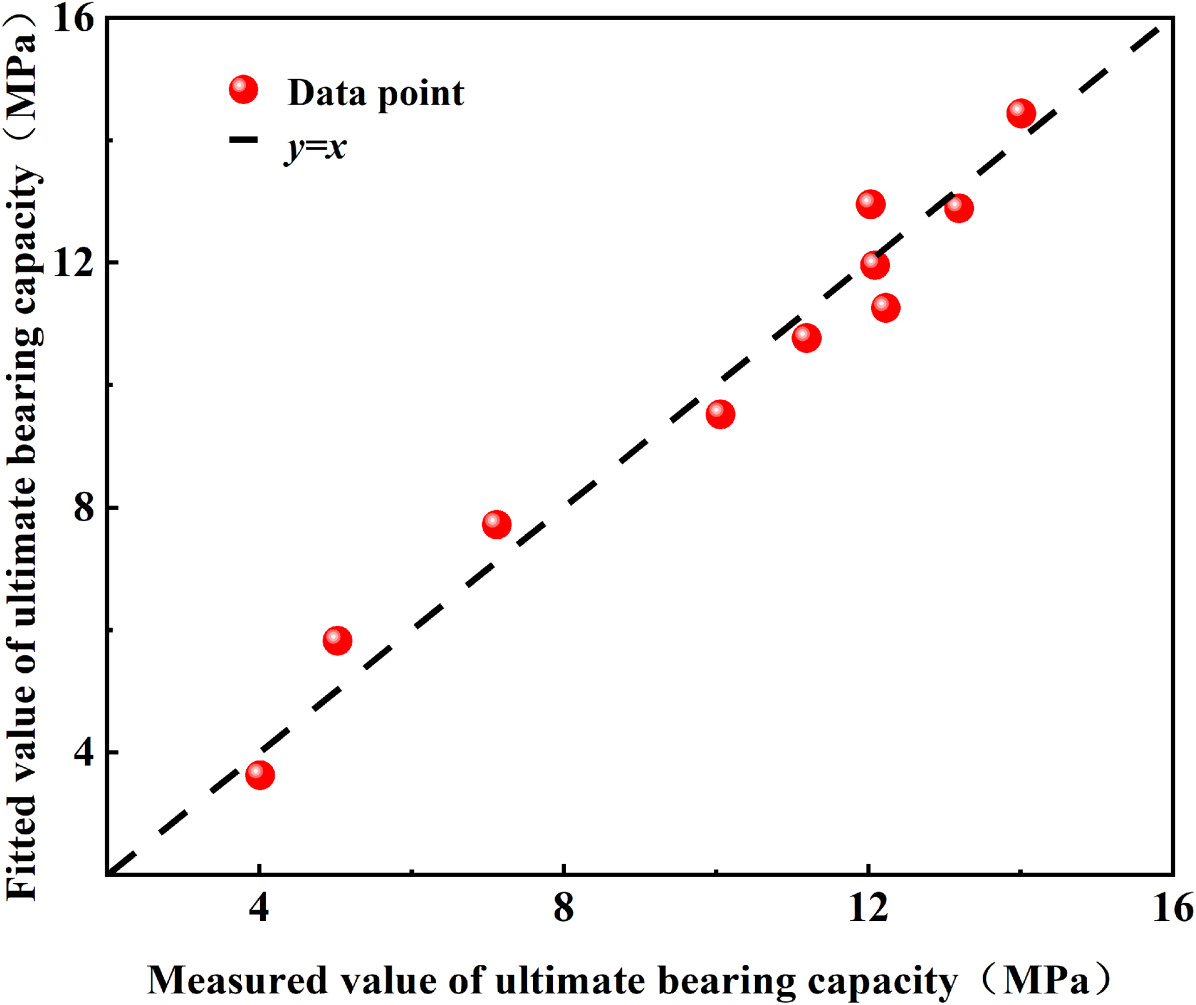
Comparison of polynomial fitting curves with different factor combinations.

As shown in Fig 10, the fitted values obtained from Equation (8) are generally distributed around the *y*=*x* line, with a coefficient of determination R^2^ = 0.97. This indicates that the proposed empirical model can accurately capture the evolution of the pile bearing capacity with respect to *β* and *h*.

**Fig 11:**
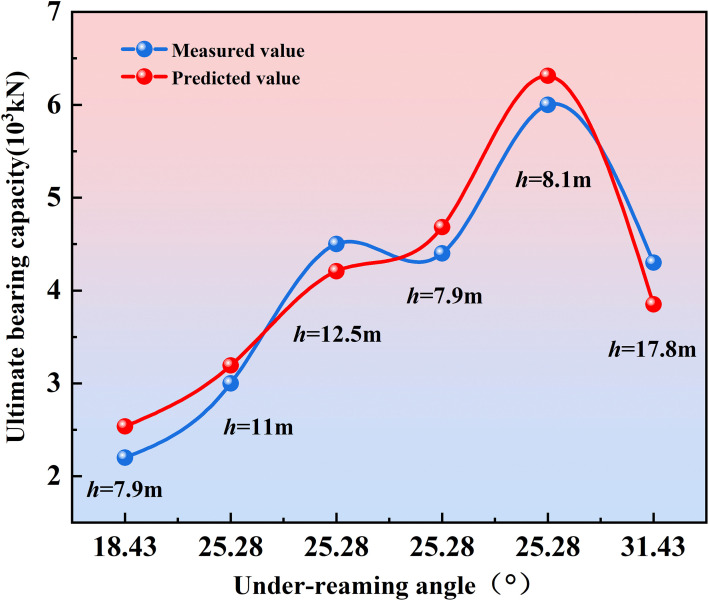
Comparison between model predictions and measured results.


To further evaluate the engineering applicability of the model, the under-reamed pile test data reported by Zhang Lei et al. [7] were selected for external validation. Based on the empirical model proposed in this study, a new empirical formula Equation (9) was obtained by re-regressing their dataset, and the predicted bearing capacities were compared with the measured values, as shown in Fig 11.

$$F=11\mathrm{.332}-1\mathrm{.679}\left(\frac{\beta }{h}\right)+\text{0}\mathrm{.236}{\left(\beta h\right)}^{0.908}$$ (9)

The results show that the predicted values from the formula exhibit high consistency with the experimental measurements reported in the literature, with a determination coefficient R^2^ = 0.93 and a prediction error ranging from 4.94% to 13.25%. This confirms that the empirical model established in this study, through regression-based inversion, can adequately describe the nonlinear algebraic relationship between the bearing capacity of under-reamed piles and the combined effects of under-reaming angle and embedded depth.

### 3.7 Analysis of axial force distribution along the pile shaft

Based on the strain data collected from the strain gauges embedded in the pile shaft, the axial force at each measurement point can be calculated using the following formula:


$$p=E\times A\times \varepsilon_{i}$$ (10)

where *p* is the axial force at the *i*-th strain gauge (kN); $\varepsilon_{i}$ is the strain value at the *i*-th strain gauge; *E* is the elastic modulus of the pile material (kPa); and *A* is the cross-sectional area of the model pile (m^2^).

**Fig 12:**
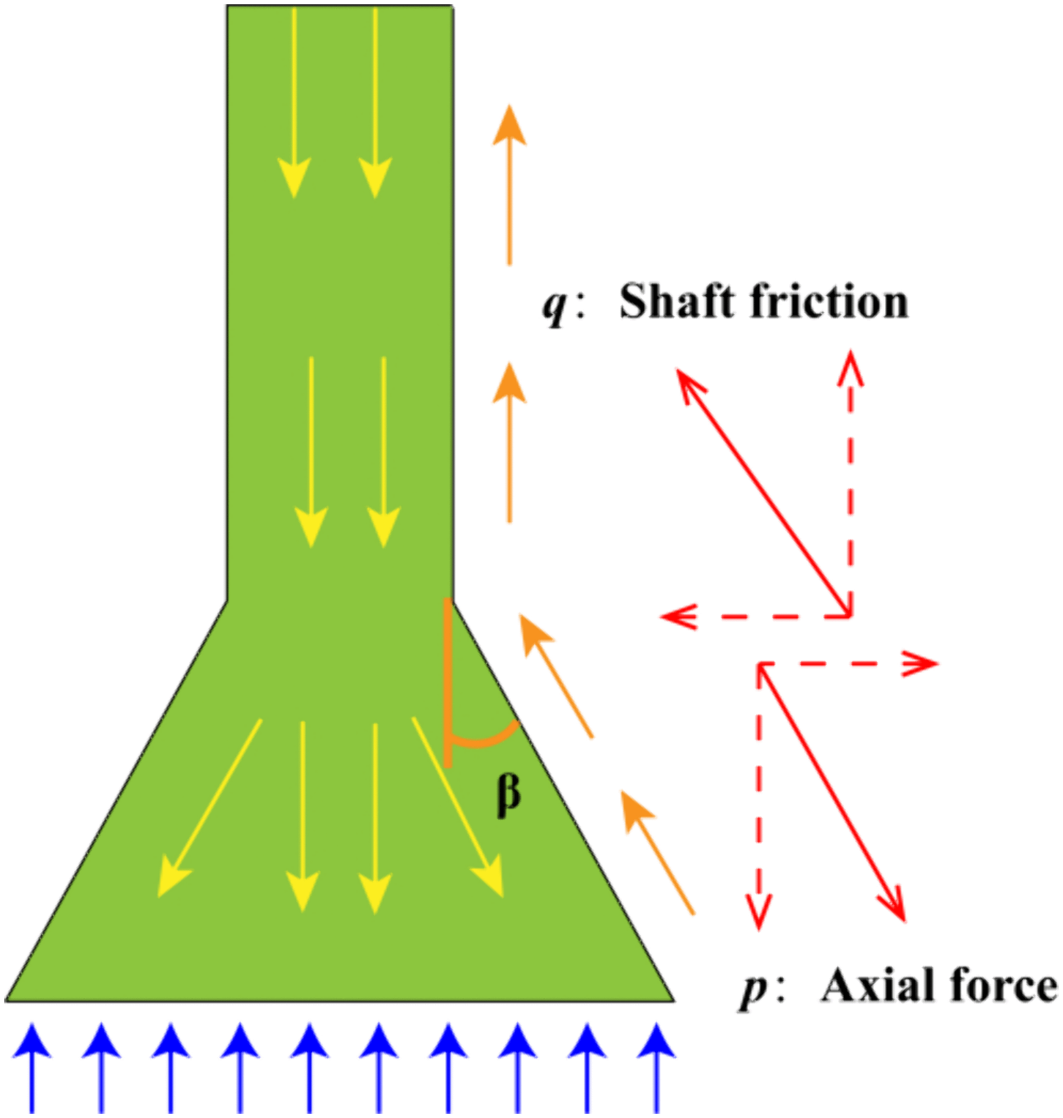
Schematic of axial force distribution along the pile shaft. (1) *β* = 0°. (2) *β* = 5°. (3) *β* = 10°. (4) *β* = 15°. (5) *β* = 20°. (6) *β* = 25°.


The axial force distribution in the under-reamed section of the pile is shown in Fig 12. The axial force calculation formula for the under-reamed section of the pile can be derived by rearranging Equation (10) [33]:

$$p^{\prime }=E\times A\times \varepsilon_{i}\times cos\beta$$ (11)

**Fig 13:**
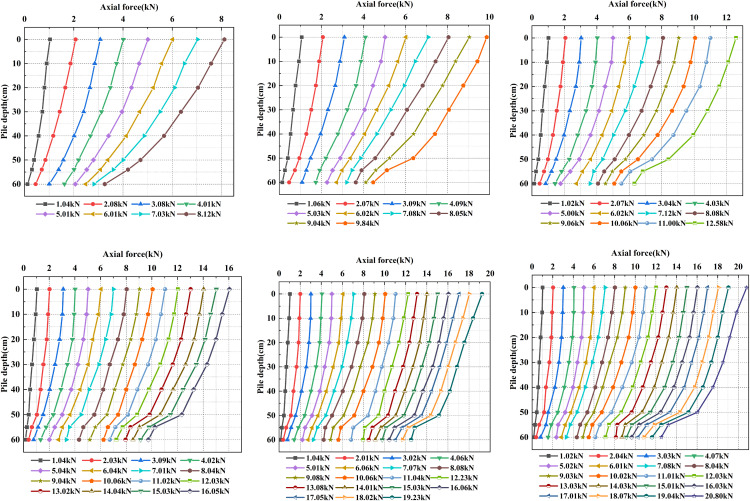
Axial force along the pile shaft at 60 cm embedment depth under different under-reaming angles. (1) *β* = 15°, *h* = 70 cm. (2) *β* = 20°, *h* = 70 cm. (3) *β* = 15°, *h* = 80 cm. (4) *β* = 20°, *h* = 80 cm.

Based on Equation (10) and (11), the axial force curves for piles with different under-reaming angles under vertical loading were calculated and plotted. Fig 13 presents the axial force distribution of under-reamed piles with an embedment depth of 60 cm under six working conditions.

As shown in Fig 13, under the same embedment depth, the axial force at each measuring point increases with increasing top load. At a constant top load, the axial force decreases progressively with depth, indicating that the load is gradually transferred to the surrounding soil through shaft resistance. The horizontal offset of the curve directly reflects the magnitude of axial force reduction along the pile depth, which represents the degree of shaft resistance mobilization.

When *β =* 0°, the axial force decreases approximately linearly with depth. Before reaching the ultimate bearing capacity, the reduction is relatively mild, but once the ultimate load is exceeded, the decrease becomes more pronounced. For 5° and 10° piles, a more substantial reduction in axial force occurs in the under-reamed section (50 ~ 60 cm), suggesting that more load is transferred through shaft resistance, alleviating the concentrated stress at the pile toe. As *β* increases to 15° and 20°, this feature becomes more prominent. The axial force reduction remains uniform in the upper shaft but significantly increases in the under-reamed section, showing that the enlarged base enhances shaft resistance, promotes deeper load transfer, and improves the ultimate bearing capacity. However, when *β* reaches 25°, the axial force decreases too rapidly in the under-reamed section, resulting in insufficient end bearing resistance mobilization and localized soil failure.

**Fig 14:**
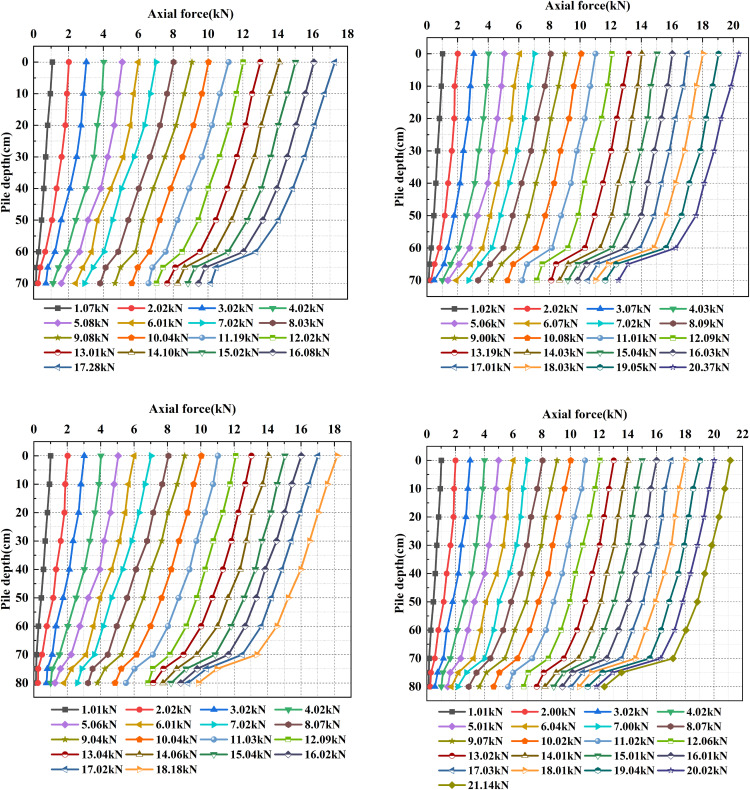
Axial force along the pile shaft under different combinations of under-reaming angles and embedment depths.

As shown in Fig 14, regarding the embedment depth effect, under the same under-reaming angle, increasing the embedment depth from 60 cm to 80 cm significantly affects the axial force transfer pattern. As the embedment depth increases, the contact area between the pile and surrounding soil enlarges, enhancing the mobilized shaft resistance. Consequently, more load is dissipated along the shaft, and the residual axial force at the pile toe decreases notably. Specifically, the axial force-depth curve becomes flatter in the upper portion of the pile, while a sharper drop is observed near the under-reamed section. For piles with a 20° angle, the axial force reduction in the under-reamed section is greater than that of 15° piles, indicating stronger pile-soil interlock and shaft resistance contribution.

This behavior demonstrates that increasing embedment depth strengthens pile-soil interaction, promotes a more uniform load transfer pattern, and mitigates excessive concentration of end bearing stress. Therefore, a moderate increase in embedment depth can effectively enhance overall bearing capacity, improve load-sharing coordination, and reinforce the composite bearing behavior of the pile-soil system.

### 3.8 Analysis of shaft resistance resistance

To further investigate the load-sharing mechanism of under-reamed piles, the distribution characteristics of shaft resistance resistance were analyzed based on the axial force data along the pile shaft. The shaft resistance resistance between two adjacent strain gauges can be calculated using the following formula:


$$q=E\times A_{p}\times \left(\varepsilon_{i}-\varepsilon_{j}\right)$$ (12)

where *q* is the shaft resistance resistance (kN), *E* is the elastic modulus of the pile material (kPa), *A*_*p*_ is the cross-sectional area of the pile (m^2^), $\varepsilon_{i}$ and $\varepsilon_{j}$ are the strain values at the *i*-th and *j*-th strain gauges, respectively.

The formula can be further modified for the under-reamed section to derive the corresponding shaft resistance resistance equation, as shown in Equation (12).


$$q^{\prime }=E\times A_{p}\times \left(\varepsilon_{i}-\varepsilon_{j}\right)$$ (13)

Using Equations (12) and (13), the shaft resistance resistance and end bearing capacity at the ultimate load state under different working conditions were calculated, as summarized in Table 5.

**Table 5:** Distribution of end-bearing and shaft resistance resistance of under-reamed piles.

Figure	Pile top load (kN)	End bearing resistance (kN)	shaft resistance resistance of the uniform section (kN)	shaft resistance resistance of the under-reamed section (kN)	Proportion of end bearing resistance (%)	Proportion of shaft resistance resistance of the uniform section (%)	Proportion of shaft resistance resistance of the under-reamed section (%)
1#	4.01	1.62	1.99	0.40	40.4	49.63	9.98
2#	5.03	2.27	2.25	0.51	45.13	44.73	10.14
3#	7.12	3.59	2.79	0.74	50.42	39.19	10.39
4#	10.06	6.24	2.65	1.17	62.03	26.34	11.63
5#	12.23	7.96	2.74	1.53	65.09	22.4	12.51
6#	12.03	7.12	3.33	1.58	59.19	27.68	13.13
7#	11.19	6.59	3.57	1.03	58.89	31.9	9.2
8#	12.09	6.85	4.20	1.04	56.66	34.74	8.6
9#	13.19	8.12	3.79	1.28	61.56	28.73	9.7
10#	14.01	8.24	4.47	1.30	58.82	31.91	9.28

**Fig 15:**
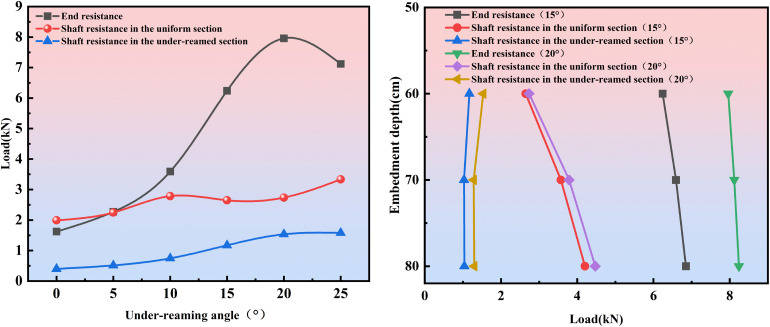
Evolution of end-bearing and shaft resistance resistance of under-reamed piles under different under-reaming angles and embedment depths. (1) Cases with different under-reaming angles. (2) Cases with different embedment depths.

As shown in Fig 15, when the under-reaming angle gradually increases from 0° to 25°, the end bearing resistance of the pile first increases and then decreases, reaching its maximum value of 7.96 kN at *β = *20°. The shaft resistance resistance of the uniform section generally exhibits a “first increase-then decrease-then increase again” trend, although its fluctuation amplitude is relatively small. The shaft resistance resistance of the under-reamed section increases continuously within the range of 0° ~ 20°, but its variation becomes insignificant between 20° and 25°.

It can be observed that as the under-reaming angle *β* increases from 0° to 20°, the end-bearing resistance shows the most significant growth, increasing from 1.62 kN to 7.96 kN, corresponding to an improvement of approximately 391%. In contrast, the total shaft resistance (including both the shaft resistance of the uniform section and the shaft resistance of the under-reamed section) increases from 2.39 kN to 4.27 kN, corresponding to an increase of about 79%. Consequently, the ultimate bearing capacity increases from 4.01 kN to 12.23 kN (an improvement of approximately 205%). This indicates that the enhancement in end-bearing resistance is the primary driving force for the overall increase in bearing capacity, whereas the shaft resistance provides a stable and synergistic supporting contribution.

The test results under different embedment depths further highlight the significant influence of the under-reaming angle on the bearing behavior of the pile foundation. As the embedment depth increases from 60 cm to 70 cm and then to 80 cm, the increments in end bearing resistance for the 15° under-reamed pile are 5.61% and 3.95%, reflecting a reduction of 1.66% in the growth rate. Under the 20° condition, the corresponding increments are 2.01% and 1.48%, with a decrease of 0.53% in the growth rate. Although the end bearing resistance continues to increase with greater embedment depth, its rate of growth gradually slows as the under-reaming angle rises. The shaft resistance of the uniform section is even more sensitive to changes in embedment depth. For the *β* = 15° condition, the increments are 34.72% and 17.65%, indicating a reduction of 17.07% in the growth rate; for *β* = 20°, the increments are 38.32% and 17.94%, showing a decrease of 20.38%. Therefore, while deeper embedment enhances the shaft resistance of the uniform section as the under-reaming angle increases, the rate of improvement diminishes progressively.

In contrast, the contribution of the shaft resistance resistance of the under-reamed section gradually weakens with increasing embedment depth. Under *β = *15° condition, the resistance first decreases by 11.97% and then increases by 0.97%, resulting in an overall reduction of about 11%. For *β = *20° condition, it first decreases by 16.34% and then increases by 1.56%, leading to a total reduction of 14.78%. These results indicate that as the under-reaming angle and embedment depth increase, the end bearing resistance grows slowly, the contribution of the uniform section’s shaft resistance becomes more significant, while that of the under-reamed section gradually diminishes.

**Fig 16:**
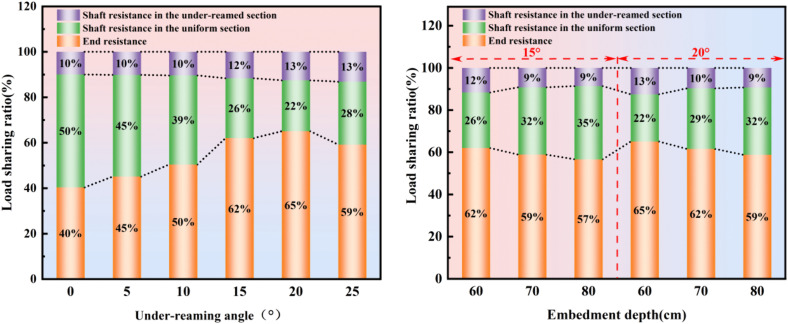
Load sharing ratio of end-bearing and shaft resistance resistance of under-reamed piles.

For foundations resting on weathered rock bearing layers, it is essential to fully mobilize end bearing resistance in conjunction with shaft resistance to achieve optimal load transfer. As shown in Fig 16(1), with the under-reaming angle increasing from 0° to 25°, the end bearing resistance sharing ratio first increases and then decreases. Compared with the 0° condition, the end bearing resistance sharing ratio gradually rises with larger under-reaming angles. For *β* = 15° and *β* = 20° conditions, the end bearing resistance sharing ratio exceeds 60%, indicating a strong bearing advantage. However, when the angle increases to 25°, although the shaft resistance sharing ratio of the under-reamed section slightly increases, its growth relative to the 20° case is only 0.62%, while the end bearing resistance sharing ratio decreases by 5.9%. This demonstrates that optimizing the under-reaming angle is a more efficient means of improving bearing performance.

Under small under-reaming angles (0° ~ 10°), the load is mainly transferred along the pile shaft, and side friction bears the majority of the load (as shown in Table 5 and Fig 16). The proportion of end resistance accounts for only about 40% ~ 50%. As the under-reaming angle increases, a pronounced stress concentration develops around the “shoulder” of the under-reamed section, which enlarges the stress diffusion angle at the pile base and enables the end resistance to be fully mobilized. The synergistic contribution between side friction and end resistance becomes significantly enhanced. When the under-reaming angle reaches 15° ~ 20°, the proportion of end resistance rises to more than 60%, and the load-transfer mechanism of the pile-soil system shifts from a “shaft resistance-dominated” mode to an “end-resistance-dominated” mode, exhibiting a distinct “fan-shaped compaction” pattern.

Comparing the performance of *β* = 15° and *β* = 20° under-reamed piles at different embedment depths (Fig 16(2)), it can be observed that as the embedment depth increases, both the end bearing resistance and the shaft resistance sharing ratio of the under-reamed section decrease, whereas the shaft resistance sharing ratio of the uniform section gradually increases. This phenomenon is mainly because increasing embedment depth leads to a longer contact interface between the pile and the soil and a larger lateral surface area of the pile, which enhances the shaft resistance of the uniform section. Consequently, the load transfer along the pile becomes more evenly distributed, and the proportion of shaft resistance in the overall bearing system rises, relatively weakening the contributions of the end bearing and the under-reamed section.Under the same embedment depth, the end bearing resistance and under-reamed section shaft resistance sharing ratios for *β* = 20° condition are higher than those for *β* = 15° condition, but the difference becomes smaller as embedment depth increases. Conversely, the uniform section exhibits higher shaft resistance sharing under *β* = 15° condition. Although deeper embedment generally enhances the shaft resistance sharing ratio of the uniform section, the extent of improvement is significantly influenced by the under-reaming angle.

Further comparison indicates that the enhancement in end bearing resistance sharing ratio due to increasing the under-reaming angle (from 15° to 20°) is far greater than that produced by increasing embedment depth (from 60 cm to 80 cm). In the present study, the under-reaming angle is thus identified as the dominant factor influencing the load-sharing mechanism, surpassing the effect of embedment depth.

Overall, blindly increasing pile embedment depth reduces the end bearing resistance sharing ratio, meaning that deeper embedment does not necessarily yield better bearing performance. Selecting an appropriate under-reaming angle is a more effective way to mobilize end bearing resistance. The under-reaming angle and embedment depth exhibit a coupled control effect on the bearing behavior of under-reamed piles, but the angle design is the more critical parameter. In engineering practice, the optimal under-reaming angle should be determined first, followed by the selection of a suitable embedment depth to ensure their synergistic interaction. According to the findings of this study, under-reamed piles with angles of 15° and 20° demonstrate the most desirable bearing characteristics.

## 4 Discussion

### 4.1 Coupled control mechanism of under-reaming angle and embedment depth

The experimental results indicate that the under-reaming angle and embedment depth are two key geometric parameters governing the bearing performance of under-reamed piles. These two parameters interact within the pile-soil system, exhibiting a coupled relationship rather than acting independently.

The under-reaming angle primarily regulates the diffusion range of pile-end stress and the load transfer path, thereby determining the compaction effect of the bearing stratum and the final failure mode. The embedment depth, on the other hand, enhances the pile-soil contact area and increases lateral confining pressure, which strengthens the contribution of shaft resistance resistance to load sharing. The degree of compatibility between these two factors directly determines the synergistic efficiency between end bearing resistance and shaft resistance resistance.

When the under-reaming angle and embedment depth are properly matched (as in this study, where an under-reaming angle of 15° ~ 20° combined with an embedment depth of 60 ~ 70 cm), a stable and continuous load transfer path is formed among the “pile body-under-reamed section-bearing stratum.” Under these conditions, the end bearing resistance can be fully mobilized, while the shaft resistance and end bearing resistance act in coordination. The soil at the pile toe exhibits a regular “fan-shaped compaction” failure pattern, and the pile foundation as a whole demonstrates excellent bearing performance and deformation compatibility.

The test results show that increasing the under-reaming angle can markedly improve end resistance; however, when the angle becomes excessively large (e.g., *β = *25°), strong stress concentration occurs at the pile shoulder, resulting in a reduction in ultimate bearing capacity. The strain and axial-force distributions indicate that although the overall strain increases with the under-reaming angle, the strain decrease at the pile shoulder is most significant at *β = *25°, reflecting local instability at this location. The load-displacement curves further show lateral deviation of the under-reamed section, and the pile failure morphology (Fig 4(6)) reveals circumferential spalling and local crushing near the shoulder. This indicates that the force-transfer path between the under-reamed section and the upper uniform-diameter segment is disrupted, thereby limiting the further mobilization of end resistance.

### 4.2 Implications for engineering design and practice

Based on the above mechanism, engineering design should prioritize optimizing the under-reaming angle, complemented by a reasonable embedment depth. The improvement in bearing capacity achieved solely by increasing embedment depth is rather limited. Maintaining the under-reaming angle within a reasonable range of 15° ~ 20° not only enhances pile-end bearing resistance but also optimizes the load transfer path, thereby improving the efficiency and stability of the pile foundation.

For projects in which weathered rock serves as the bearing layer and the overlying soil mainly consists of silty clay, the under-reaming angle can be used to control the mobilization of end bearing resistance and the extent of stress diffusion. Embedment depth should serve as an auxiliary factor to adjust shaft resistance resistance and improve pile-soil interlocking. It is recommended to determine the “critical matching range” of these parameters through experimental tests or numerical simulations to prevent adverse effects such as shoulder voids or significantly increased construction difficulty caused by an excessively large under-reaming angle.

In summary, the rational matching of under-reaming angle and embedment depth is the key to optimizing the bearing performance of under-reamed piles. Their coordinated design can fully exploit the composite reinforcement effect of end bearing resistance and shaft resistance resistance, providing essential theoretical support for parameter optimization and engineering application of under-reamed pile foundations.

## 5 Conclusions

Based on a semi-model static load testing system, this study systematically investigated the vertical bearing performance of large-diameter under-reamed piles under different under-reaming angles and embedment depths. The load transfer law, settlement deformation characteristics, and failure mechanism of the soil at the pile toe were revealed, providing a reference for the optimization of design parameters and engineering application of under-reamed piles. The main conclusions are as follows:

1. With the increase of under-reaming angle and embedment depth, the ultimate bearing capacity of the under-reamed pile increases overall, among which the effect of under-reaming angle is the most significant. When the under-reaming angle is 20°, the bearing capacity increases by up to 204.99%. Although increasing the embedment depth can further enhance the bearing capacity, the growth rate gradually decreases. Considering both bearing performance and structural stability, the optimal under-reaming angle range is 15° ~ 20°. When the under-reaming angle exceeds this range, stress concentration at the “shoulder” area tends to cause pile-soil separation, limiting further improvement in bearing capacity.
2. The axial force of the uniform section of the pile decreases approximately linearly with the increase of embedment depth, while the attenuation of axial force in the under-reamed section becomes more significant. As the under-reaming angle increases, the load sharing ratio of the pile toe resistance shows a trend of “first increasing and then decreasing,” and the load transfer mechanism transforms from being dominated by shaft resistance resistance to being dominated by end bearing resistance. Under the optimal under-reaming angle, the load sharing ratio of the pile toe resistance exceeds 60%. Although the proportion of shaft resistance resistance in the under-reamed section is relatively small, it plays a key role in realizing the coordinated transfer between end bearing and shaft resistance resistance.
3. With the increase of under-reaming angle, the failure mode of the rock or soil at the pile toe changes from the “penetrative shear” mode of the uniform-section pile, with a failure range of about 1.25D, to the “fan-shaped compaction” mode under the optimal under-reaming angle, with a failure range of about 4D. When the under-reaming angle becomes too large, the failure mode evolves into an unstable “bulging-collapse” pattern. This evolution law provides a reliable basis for identifying the working state and instability characteristics of under-reamed piles.

## Supporting information

S1 Data Raw experimental data corresponding to Figs 5–16 of this article. The Excel file contains the original numerical data used to generate the figures, including load–displacement curves, ultimate bearing capacity, axial force distribution along the pile shaft, and the evolution of end-bearing and shaft resistance under different under-reaming angles and embedment depths. (XLSX)

S2 Data Original Origin project files corresponding to Figs 5–16, used for figure generation and data analysis. These files include the original datasets and plotting settings and are provided as a ZIP archive. (ZIP)
